# Recent Studies in Rabies

**Published:** 1906-07

**Authors:** James N. Brawner

**Affiliations:** Atlanta, Ga.; Physician in Charge of Georgia Pasteur Institute


### RECENT STUDIES IN RABIES.

BY JAS. N. BRAWNER, M.D., ATLANTA, GA.

Physician in Charge of Georgia Pasteur Institute.

Since Pasteur's memorable work on rabies, demonstrating the constant occurrence of the virus in the nervous system and the means of attenuating it so that preventive inoculations might be made, numerous investigators have studied this disease in every detail. Especial attention has been paid to the pathology and every portion of the nervous system has been minutely scrutinized by the best histologists in search of specific lesions. Other workers have been especially active in trying to improve on the preventive inoculations of Pasteur or to get an effective serum. One or two have been equally active in trying to disprove the existence of such a disease communicable to man, and have written many articles setting forth their claims. Those who deny the existence of rabies are fast disappearing and it is now rare to see a physician skeptical on the subject.

While an immense amount of work has been done to improve on the preventive treatment now used at all the Pasteur institutes, but little has been accomplished. Various means have been used to attenuate the virus and many attempts have been made to obtain a serum that would be effective after symptoms develop, but practically no improvements have been made on the method of treatment first demonstrated by Pasteur, excepting, perhaps, that much larger doses than formerly are now used.

The pathology of rabies is interesting from many standpoints and important results have been obtained from recent investigations along this line. One of the most practical questions has been, can a specific lesion or micro-organism be found so that a rapid and positive diagnosis may be made from a microscopical examination of the tissues. The lack of a means for rapid diagnosis in cases of suspected rabies in animals has been long felt, as patients are often put to unnecessary work and delay awaiting the result of the inoculation of rabbits.

It is not necessary to describe the "rabic tubercle" of Babes, or the epitheloid infiltrations about the neurons, first mentioned by Van Gehutchten and Nelis; but we will consider more in detail those structures more recently described, now commonly known as "Negri bodies."

In March, 1903, Dr. A. Negri, of the University of Pavia, announced the discovery of certain bodies in the central nervous system of animals dead of rabies which he believed to be the specific germ of this disease. This announcement, coming from Dr. Golgi's assistant, and one perfectly familiar with microscopical appearance of both normal and pathologic nerve tissue, at once attracted attention. Since that time numerous investigators, especially the Italians, have confirmed his results.

In August, 1903, Dr. Negri made a further report, stating, to that time he had examined the nervous systems of over one hundred animals suffering from rabies and various other diseases, and found the bodies only in the animals proven to be rabid by the inoculation test. In two instances where rabies existed the structures could not be demonstrated.

Later Bertarelli and Volpino found the bodies described by Negri in the brain of a boy who died of hydrophobia. Bertarelli, summarizing the work done in Italy by Negri, Volpino, Abba,

Bermans and himself, states that in more than a thousand tests the bodies were found in all cases of rabies except three, and were never found in animals free from the disease, as determined by the inoculation of rabbits.

Poor, in New York, after twenty-three examinations, confirmed the results of the Italians.

The structures described by Negri are usually found in the cytoplasm of nerve cells or the proximal ends of their processes, but not in the nucleus, though they may be in contact with it. He believes them to be protozoa. They occur most frequently as inclusions of the large pyramidal cells of the hippocampus major, Purkinjé cells of the cerebellum, the large cells of the spinal ganglia, spinal cord, pons and occasionally the pyramidal cells of the cerebral cortex. As a rule they are much more numerous in the hippocampus major than in other portions of the nervous system, though this is not always the case, as they may be absent here and numerous in the spinal cord or cerebellum. Negri states that when rabies is produced by inoculation into the sciatic nerve the bodies are found chiefly in the spinal cord and ganglia, rarely in the brain. When the inoculation is on the face, they are more abundant in the gasserian ganglia, hippocampus major, and cerebellum. There seems to be a difference of symptoms corresponding to the distribution of the bodies. Ordinarily, when the disease is acquired in the natural manner they are larger and more numerous in the hippocampus major. This is probably due to the fact that most bites communicating the disease are inflicted on the head or face.

As usually found, Negri bodies are from four to six microns in diameter. Occasionally, especially early in the disease, they are very small, barely visible with a 1-12 oil immersion. At other times they are large, measuring twenty-five microns in length. The larger forms are more frequently found in laboratory rabies and in the natural form closest to the site of inoculation.

They are round or oval in shape, occasionally irregular or elongated. When seen in fresh, unstained tissue, teased by a dilute of acetic acid solution, they seem to consist of a non-granulated substance of hyaline appearance. In the center of the larger forms bright spots may be seen, surrounded by a band of homogenous protoplasm. In specimens stained with eosin and methylene blue, they appear as dark red structures imbedded in the blue protoplasm of the nerve cells. They show up black in specimens stained with osmic acid.

To demonstrate these bodies, fix a small piece of each the hippocampus major and cerebellum in a saturated solution of corrosive sublimate, or of Zenker's fluid, dehydrate in absolute alcohol for six or eight hours, and section in paraffin. The sections should be thin and uniform. Fix upon a slide with Mayer's albumen, treat with a one-half per cent. aqueous solution of eosin for five minutes, wash in water and counter stain with a one-half per cent. aqueous solution of methylene blue for five minutes. Rinse in water, dehydrate in absolute alcohol, clear in xylol and mount is balsam. In specimens thus prepared, the Negri bodies stand out very distinctly as dark red structures, imbedded in the blue cytoplasm of nerve cells. As a rule, but one body is found in a cell, though occasionally two, three, or even four, may be found. The smaller bodies appear homogeneous in structure, but the larger ones contain a blue central mass resembling a nucleus.

While most observers believe these bodies to be the causative factor in rabies, all admit that positive proof is lacking. The definite morphology of the structure and the constancy of their occurrence, tend to substantiate such a belief. Three hypotheses may be offered to explain their occurrence: (1) Degenerations, or artefacts of the cell protoplasm; (2) Phagocytosis, that is, the nerve cells attracting and enveloping certain other normal histological structures, as red blood corpuscles; (3) The specific micro-organism of the disease.

As to the first hypothesis, Negri states that artefacts can not be made by any method of preparation to simulate these bodies, and his conclusions are corroborated by all observers so far as I am able to determine. The question of degenerations should be carefully considered. While it is the general opinion that no form of metamorphosis of the cell protoplasm could account for structures so constantly found in certain cells, so definite in shape and uniform in staining reaction, yet the possibility of the toxin causing a specific form of degeneration should be kept in mind.

Poor states that in a case of tetanus minute eosinophilic granules were found in the Purkinjé cells of the cerebellum, but to an experienced eye they would not be mistaken for Negri bodies. Nerve cells in all stages of degeneration show no such structures as are found in rabies. Negri bodies retain their characteristics in cells in all stages of degeneration and Negri states that they even resist the putrefactive process.

The theory of phagocytosis is less tenable than that of specific degeneration. To the inexperienced, erythrocytes are more likely to be mistaken for Negri bodies than any other histological structure, as they are about the same size, though usually larger, and are eosinophilic in reaction. This fact suggested the hypothesis that Negri bodies were red blood corpuscles phagocytized by nerve cells. To me this view is untenable for many reasons. While both Negri bodies and erythrocytes stain red, the former are of a darker color. In fresh preparations the bodies are hyaline and have not the slightest resemblance to red blood cells, and in sections stained with osmic acid they show up black, while the latter remain unstained. It is also impossible to conceive of a phagocytized red blood corpuscle increasing in size and developing a nucleus. Phagocytized red blood corpuscles in the spleen show no resemblance of Negri bodies, but are found as fragments and in various stages of degeneration.

Both direct and indirect evidence in favor of these structures being the specific germs of the disease may be said to exist. As stated by Bertarelli: "In more than a thousand tests the Negri bodies were never found in animals free from rabies as determined by inoculation of rabbits. On the other hand, they were always found in infected animals with three exceptions, and in these only a part of the nervous system had been examined." It may be said that in all cases of rabies, if every part of the nervous system were examined, these bodies would invariably be found. All observers agree that Negri bodies are never found in other diseases, even in those where the degenerative changes in the nerve cells are pronounced. Judging from analogy, we must admit that rabies is caused by a specific micro-organism, and from its behavior to heat, chemicals and culture media, we are probably justified in believing it to be a protozoan.

The virus is destroyed in a few minutes at a temperature of 110° F. Two per cent. carbolic acid renders it inert in a few seconds, and it is rapidly destroyed by any of the weaker antiseptics. It retains its virulence for weeks or months in neutral glycerine. Glycerine that is slightly acid destroys the virus. It can not be grown on any known culture medium.

Like many protozoan diseases, it is communicated through the saliva. We now know that malaria, mountain fever, cattle fever, and probably yellow fever, are protozoan diseases, and are all communicated through the saliva of infected mosquitoes, ticks, etc. While it is true that the virus of rabies does not pass through an intermediate host, but directly from animal to animal, yet the fact that it is eliminated by way of the salivary glands is suggestive of the protozoan nature of the organism. So, from indirect evidence in our possession, the specific germ of rabies is probably a protozoan, and as Negri bodies resemble protozoa, we are justified in assuming them to be the causative factor in this disease.

Whether or not we regard the bodies discovered by Negri as the specific cause of rabies, their value as a means of rapid diagnosis is now generally recognized. When the brain of an animal is examined and Negri bodies are found, the evidence is considered positive and a diagnosis of rabies is made. More than one thousand tests, confirmed by the inoculation of rabbits, have now been reported, and not a single exception has been found. At the Georgia Pasteur Institute, within the last eighteen months, I have demonstrated Negri bodies in forty-two dogs and three cats, and in every instance the rabbits inoculated died of typical rabies. In three cases where the inoculation test resulted positively, no bodies were found, though only the hippocampus major was examined and the animals were killed in the first stages of the disease. In fifteen dogs and two cats, proven not to be rabid, no such structures could be demonstrated.

In conclusion, it may be stated that we now have a reliable method by which the diagnosis of rabies in animals can be made in a few hours. It has many advantages over the old method, the inoculation of rabbits. Much time is saved and the patient relieved of much suspense. At present I always inoculate one or two rabbits to corroborate Negri's test, and if the bodies can not be found rabbits should invariably be inoculated to prove conclusively the absence of rabies. Dr. Negri has given us a most valuable diagnostic method, and it is the consensus of opinion that he has also discovered the specific micro-organism of rabies.

